# Discovery of N-Containing (-)-Borneol Esters as Respiratory Syncytial Virus Fusion Inhibitors

**DOI:** 10.3390/ph15111390

**Published:** 2022-11-11

**Authors:** Anastasiya S. Sokolova, Olga I. Yarovaya, Lana V. Kuzminykh, Anna A. Shtro, Artem M. Klabukov, Anastasia V. Galochkina, Yulia V. Nikolaeva, Galina D. Petukhova, Sophia S. Borisevich, Edward M. Khamitov, Nariman F. Salakhutdinov

**Affiliations:** 1 N.N. Vorozhtsov Novosibirsk Institute of Organic Chemistry SB RAS, Lavrentiev Ave., 9, 630090 Novosibirsk, Russia; 2 Smorodintsev Research Institute of Influenza, Prof. Popov St., 15/17, 197376 Saint-Petersburg, Russia; 3 Laboratory of Physical Chemistry, Chemistry Institute of the Ufa Federal Research Center, Octyabrya Pr., 71, 450054 Ufa, Russia

**Keywords:** (-)-borneol, virus entry inhibitor, surface F protein, molecular docking

## Abstract

Respiratory syncytial virus (RSV) causes acute respiratory infections, thus, posing a serious threat to the health of infants, children, and elderly people. In this study, we have discovered a series of potent RSV entry inhibitors with the (-)-borneol scaffold. The active compounds **3b**, **5a**, **5c**, **7b**, **9c**, **10b**, **10c**, and **14b** were found to exhibit activity against RSV A strain A2 in HEp-2 cells. The most active substances, **3b** (IC_50_ = 8.9 μM, SI = 111) and **5a** (IC_50_ = 5.0 μM, SI = 83), displayed more potency than the known antiviral agent Ribavirin (IC_50_ = 80.0 μM, SI = 50). Time-of-addition assay and temperature shift studies demonstrated that compounds **3b**, **5a**, and **6b** inhibited RSV entry, probably by interacting with the viral F protein that mediated membrane fusion, while they neither bound to G protein nor inhibited RSV attachment to the target cells. Appling procedures of molecular modeling and molecular dynamics, the binding mode of compounds **3b** and **5a** was proposed. Taken together, the results of this study suggest (-)-borneol esters to be promising lead compounds for developing new anti-RSV agents.

## 1. Introduction

Increasing outbreaks of acute respiratory infections, mostly caused by viruses with epidemic or pandemic potential, pose a significant threat to public health. The most common viruses causing a large proportion of acute respiratory infections are influenza viruses, human rhinovirus, respiratory syncytial virus (RSV), adenovirus, and human coronavirus [1]. The COVID-19 pandemic, caused by severe acute respiratory syndrome coronavirus 2 (SARS-CoV-2), has resulted in more than 518 million confirmed cases and more than six million deaths globally [2]. The influenza virus has raised alarming concerns worldwide. Millions of people have died from influenza pandemics (Spanish flu in 1918, Asian flu in 1957, and swine flu in 2009) [3]. Influenza viruses, coronaviruses, and other seasonal respiratory viruses tend to cause sporadic infections or seasonal epidemics in humans. However, a pandemic may occur when a new virus with sufficient transmissibility and pathogenicity emerges. Therefore, it is of great significance to find effective drugs against infections caused by respiratory viruses. 

RSV is a negative-sense single-stranded RNA virus belonging to the family Paramyxoviridae. This virus is the most common cause of acute respiratory infections in infants and children [4]. Typically, RSV is mild and accompanied by upper respiratory symptoms, with the risk of severe RSV increased by factors such as young age (<6 months), congenital heart disease, chronic pulmonary diseases, and immunocompromised conditions [5]. Globally, infection with RSV is responsible for approximately 3.2 million hospital admissions and approximately 60,000 in-hospital deaths per year [6,7]. No specific etiotropic therapy is available, and the only compound (Synagis) designed to prevent this infection is practically inaccessible to the general population [8]. Several groups of researchers around the world are actively developing antiviral agents against respiratory syncytial infection, but none of them are yet suitable for wide use in clinical practice [9,10,11]. 

Recently, we found that (-)-borneol-based esters **I**–**IV** (Table 1) containing a saturated nitrogen ring or an acyclic tertiary nitrogen atom exhibited good anti-filovirus efficacy by affecting the glycoprotein (GP)-mediated membrane fusion process [12,13]. At the same time, in vitro results with an infectious virus and in a pseudovirus system, as well as theoretical calculations, allowed us to demonstrate that (-)-borneol esters **IV** with butyl substituents are likely to be fusion inhibitors targeting the S protein of SARS-CoV-2 [14]. Moreover, we found esters **I**–**IV** to effectively inhibit the replication of the influenza A virus [15] and esters **III** with a morpholine cycle to exhibit antiviral activity against orthopoxviruses [16]. These results indicate (-)-borneol-based esters to have broad-spectrum antiviral activity, acting on an early step of the virus life cycle, and that they should be studied as RSV inhibitors.

In this study, we expanded the library of (-)-borneol-based esters and evaluated their activity against RSV. The anti-RSV structure–activity relationship (SAR) and the antiviral mechanism of the key compounds were analyzed.

## 2. Results and Discussion

### 2.1. Chemistry

**Scheme 1:**
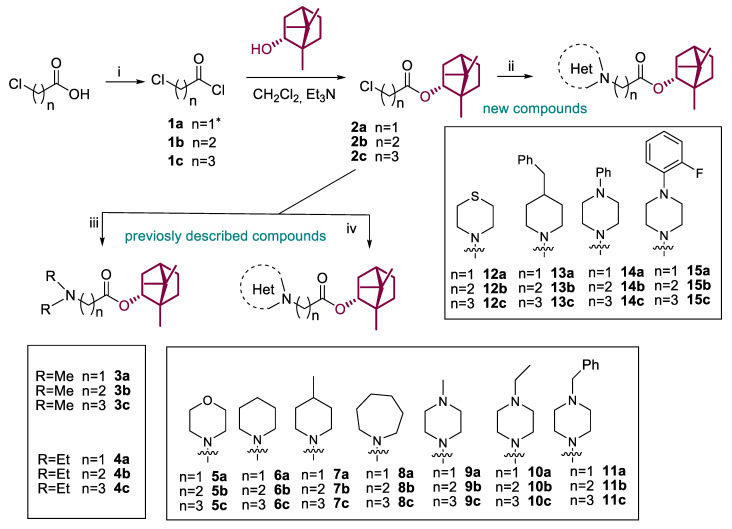
Reagents and conditions: (i) (COCl)_2_ (3 equiv), CH_2_Cl_2_, DMF (one drop), stirring at room temperature for 4 h; (ii) the corresponding amine (1.2 equiv), K_2_CO_3_ (4 equiv), KI (1 equiv), CH_3_CN, room temperature or slight heating for 12–48 h, yields 32–91%; (iii) excess of dimethylamine (40% wt. solution in ethanol) or diethylamine, K_2_CO_3_ (2 equiv), CH_3_CN, room temperature or reflux for 12 h, yields 35–53%; (iv) the corresponding amine (1.5 equiv), Et_3_N (1 equiv), CH_2_Cl_2_, room temperature or slight heating for 12–48 h, yields 32–91%. * Compound **1a** was used as a commercial reagent.

A series of (-)-borneol derivatives, 12 of them being novel, were obtained, as shown in Scheme 1. The synthesis of compounds **2**–**11a**–**c** was reported previously [12]. The synthesis of the derivatives with thiomorpholine **12a**–**c**, 4-benzylpiperidine **13a**–**c**, and N-phenylpiperazine **14**–**15a**–**c** cycles was carried out as described earlier with minor changes in the second stage. Acetonitrile was used as a solvent instead of methylene chloride. Potassium carbonate was used as a base and potassium iodine was added to activate alkyl halides **2a**–**c**. The products were isolated for column chromatography, with hexane-ethyl acetate used as the eluent.

### 2.2. SAR for the Anti-RSV Activity of the Target Compounds

All target compounds were evaluated for their in vitro antiviral activity against the RSV A strain A2 in HEp-2 cells. The IC_50_ and SI values of each compound were taken as criteria for evaluating the antiviral activity. The structures and antiviral activities of compounds **3**–**15a**–**c** are listed in Table 2.

In the series of (-)-borneol esters **3**–**4a**–**c** containing alicyclic substituents, derivative **3b**, carrying methyl groups at the nitrogen atom was found to be the most potent, with an IC_50_ value of 8.9 µM and an SI value of 111. The analogs of derivative **3b** with different linker lengths (compounds **3a** and **3c**) and the analogs with ethyl groups (compounds **4a**–**c**) turned out to be more toxic and exhibited lower activity than derivative **3b**. Within the subfamily of (-)-borneol esters **5–15a**–**c** with N-containing heterocycles, derivatives **5a-c** containing morpholine moieties showed high activity with IC_50_s of 5.0, 8.5, and 5.8 µM depending on linker length, with n = 1, 2, and 3, respectively. The morpholine derivatives **5a** and **5c**, with n = 1 and n = 3, demonstrated low toxicity, resulting in high SI values of 83 and 53, respectively. The introduction of heterocycle that is less polar than morpholine, such as thiomorpholine (derivatives **12a**–**c**) with the value of logPs in the range of 3–4, resulted in potency loss. Meanwhile, among the piperidine derivatives **6a**–**c**, with logP values also in the range of 3–4, the agent **6b** with linker length n = 2 showed moderate anti-RSV activity, with an IC_50_ value of 14.6 µM and an SI value of 22. The introduction of a methyl group at the 4-position of the piperidine cycle yielded compounds with higher logPs. Compound **7b** with linker length n = 2 showed moderate antiviral potency, with an IC_50_ value of 6.2 µM and an SI value of 26. Notably, compounds **8a**–**c,** with the azepane cycle differing from the piperidine derivatives by an additional methylene group in the cycle, turned out to be not active. The 4-methylpiperazine derivative **9c** with linker length n = 2 had high antiviral activity, with an IC_50_ value of 5.1 µM. Additionally, the 4-ethylpiperazine derivatives **10b** and **10c** (n = 2, n = 3) showed micromolar IC_50_ values and SI values of 20 and 21. It is worth mentioning that the logP values of piperazine derivatives **9**–**10a**–**c** are similar to those of active morpholine derivatives **5a**–**c**. 

As mentioned above, (-)-borneol esters act in the early stage of viral life, with surface glycoproteins being the likely targets of these compounds. Therefore, the fusion (F) glycoprotein on the RSV virion surface appears to be a likely target for synthesized (-)-borneol derivatives. The active binding site of the F protein of known inhibitors is enriched with amino acid residues containing aromatic fragments [17]. In this regard, we focused our attention on N-substituted piperazines **11a**–**c**, **14**–**15a**–**c**, and 4-substituted piperidines **13a**–**c** with aromatic rings. However, as shown in Table 2, aromatic substituents in the piperazine or piperidine cycle led to increased toxicity, with the CC_50_ values between 20.1 and 116.4 µM, excluding compound **14b** with a CC_50_ value of 365.7 µM. Additionally, compound **14b** exhibited the best antiviral activity, with an IC_50_ value of 8.1 µM and an SI value of 45. Other derivatives in this subseries of (-)-borneol derivatives showed lower SI values. It is worth noting that the high toxicity of (-)-borneol esters **11a**–**c** was observed earlier in HEK293T cells during a study of anti-filovirus activity, with CC_50_ values ranging from 30.1 to 59.4 µM [12]. The high toxicity may be due to higher lipophilicity. Highly lipophilic (logP > 5) compounds tend to bind to hydrophobic targets other than the desired one; therefore, there is an increased risk of toxicity [18].

Overall, the morpholine cycle and methyl substitutions at the nitrogen atom were the most favored structural fragments for the best antiviral activity. The introduction of a more hydrophobic moiety directly next to the aliphatic linker, such as ethylene groups, thiomorpholine, and the piperidine cycle, resulted in analogs with low potencies. Installing a piperazine cycle allowed introducing various substituents at the nitrogen atom, and several derivatives with good antiviral activity were found in this structure. Notably, the logP values for highly active compounds were in the range of 2.1–3.9, which is similar to the logP values for most F protein inhibitors in phase II clinical trials [19]. 

### 2.3. Mechanism of Action of the Key Compounds

#### 2.3.1. Time-of-Addition Assay

**Figure 1:**
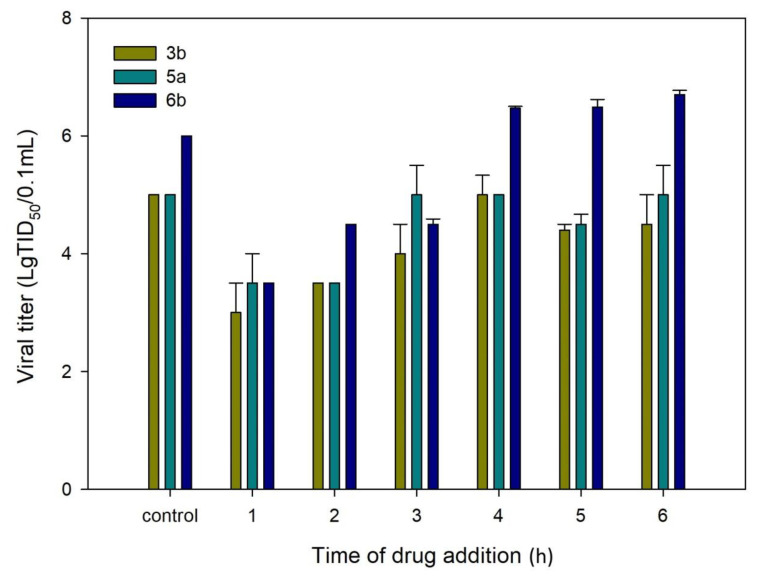
Time-of-addition assay. HEp-2 cells were infected with the RSV A strain and compounds **3b**, **5a**, and **6b** were added for different time periods after infection. Wells to which no compound was added were used as controls.

After a detailed SAR study, compounds **3b**, **5a**, and **6b** with high SI values were selected as key compounds to further investigate the mechanism of RSV replication inhibition. To identify the likely stage of the RSV life cycle that is blocked by the key compounds, we first evaluated their antiviral activity using a time-of-addition assay in HEp-2 cells. The viral life cycle of RSV follows the usual basic stages of the enveloped virus. Within the first hour after binding to the receptor, the virion penetrates the cell cytoplasm, with the subsequent release of the genome. Furthermore, within 2–20 h after penetration into the cell, the intensive replication and assembly of new viral particles occurs. After 20 h, the release of new viral particles on the cell surface begins. The time-of-addition (TOA) approach determines how long the addition of a compound can be postponed before it loses its antiviral activity. The dynamics of virus growth depending on the time of drug addition is shown in Figure 1. When added at 1 h post infection, compounds **3b**, **5a**, and **6b** were found to exhibit a significant reduction in viral titer. The antiviral activity was maintained when they were added to the cells at up to 2 h post infection. When the compounds tested were added at later time points, they lost their inhibitory activity. These data suggest that the anti-RSV activity of compounds **3b**, **5a**, and **6b** is manifested at the viral entry step, probably due to a fusion protein (F) or G-surface glycoprotein being inhibited.

#### 2.3.2. Temperature Shift Assay

**Figure 2:**
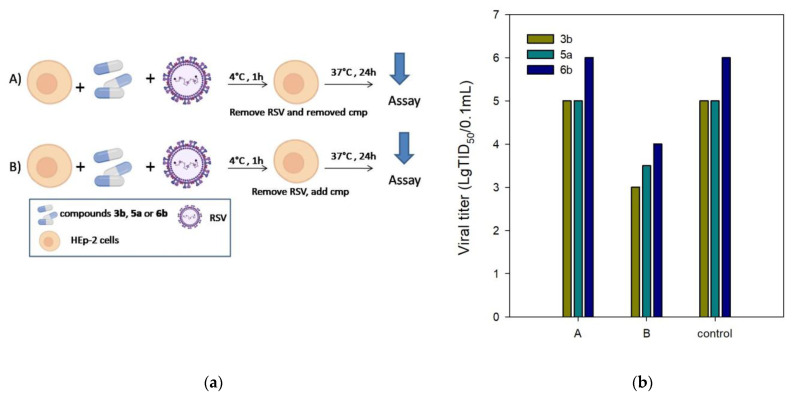
(**a**) HEp-2 cells were incubated with RSV in the presence of compounds **3b**, **5a**, and **6b** at 4 °C for 1 h and then washed to remove unbound virus and inhibitor (A), or washed to remove unbound virus and compounds added before the temperature was shifted to 37 °C (B). (**b**) Inhibition of RSV infection by the test compounds under experimental conditions (A) and (B).

The F protein plays an essential role in virus entry into host cells together with the surface glycoprotein G. The antiviral assay was performed with temperature modification to exclude the inhibition of surface glycoprotein G as a possible target of action for compounds **3b**, **5a**, and **6b**. RSV entry into host cells involves two separate events: virus–cell fusion, with glycoprotein G being the primary functional component, and cell–cell fusion, with F protein promoting the fusion of virus and host cell membranes. While RSV attachment to cells can occur at 4 °C, its fusion to the target cell membrane occurs at temperatures above 18 °C. Therefore, a compound to block virus attachment should have antiviral activity when added during virus incubation at 4 °C, followed by washing until the incubation temperature is shifted to 37 °C. Thus, to investigate the effect of compounds **3b**, **5a**, and **6b** on virus attachment, we tested the antiviral activity under experimental conditions (A) and (B) (Figure 2a). The conditions (A) were as follows: HEp-2 cells were incubated with RSV at 4 °C for 1 h to ensure the virus binding while preventing virus internalization; unbound virus and the tested compounds were washed away, and the cells were then shifted to 37 °C for the rest of the experiment. The conditions (B) involved the standard protocol for anti-RSV activity measures. As illustrated in Figure 2b, compounds **3b**, **5a**, and **6b** showed no anti-RSV activity under the experimental conditions (A). The antiviral activity was maintained in experiment (B) when the unbound virus was washed away, and the compounds tested were added to the cells. These findings confirm the possibility of post-binding events at the viral entry stage being inhibited by the compounds tested. 

### 2.4. Molecular Modeling

#### 2.4.1. Binding Site Analysis

Given the results of the time-of-addition assay, temperature shift study, and previously reported data, the F protein was suggested to be the likely target of the compounds under test. The RSV F protein is a main surface glycoprotein that mediates the fusion of the viral membrane with a host cell membrane. It is structurally similar to F proteins from other Paramyxoviridae (hydrophobic domains, heptad repeats, cysteine residues, etc.) and has the same manner of proteolytic activation, resulting in the exposition of a hydrophobic fusion peptide. The inactive precursor F0 is cleaved by the furin-like protease into an N-terminal F2 subunit and a C-terminal membrane-anchored F1 subunit carrying the fusion peptide. The fusion peptide is unique in that it is capable of causing membrane fusion even without G glycoprotein. 

The RSV F protein is used as a major target for antivirals and vaccine develop-ment because of its importance in the viral replication cycle, its conserved sequence and structure, its exposed position in the virion, and its strong immunogenicity.

**Figure 3:**
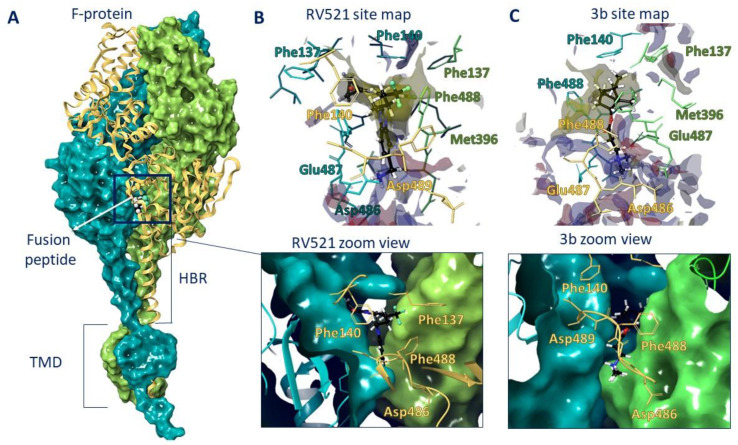
F protein trimer (**A**) with RV521 inhibitor inside. The inhibitor is located between the fusion peptide (a.a. 137–155) and HBR (a.a. 476–524). The binding site maps of RV521 (**B**) and lead compound 3b (**C**) are characterized by hydrophobic (yellow) and donor-acceptor (blue and red) features.

**Figure 4:**
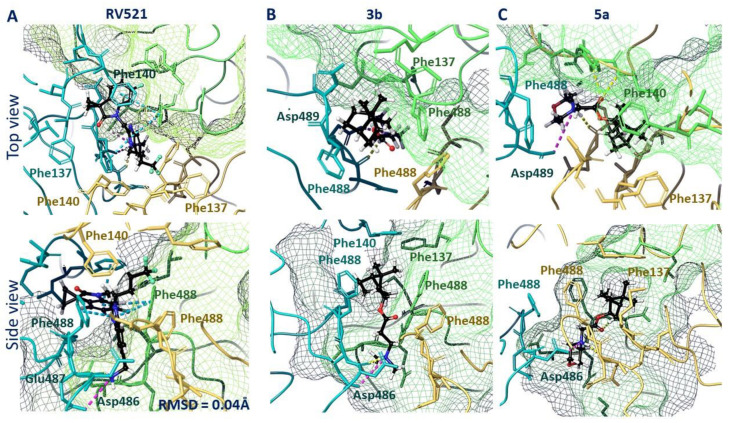
Locations of RV521 reference inhibitor (**A**), compound **3b** (**B**), and compound **5a** (**C**) in the binding site of F protein: H-bonds and salt bridges are shown as yellow and violet dotted lines, respectively, and pi–pi stacking is presented as blue dotted lines.

Therefore, we performed molecular docking studies of synthesized compounds in the active binding site of known inhibitors of the F protein. The known inhibitors of the F protein, Rilematovir [20], Sisunatovir (RV521) [19] and Presatovir [21], were found to target the same RSV F hydrophobic cavity DS-Cav1. The cavity DS-Cav1 [22] was located in the region of a fusion peptide at the N-terminus of the F_1_-subunit and the α-helix of the heptad repeat (HRB) at the C-terminus of the F_1_-subunit. To localize the binding site, the complex inhibitor RV521 bound to the F protein (PDB code 7KQD) was used [19]. The symmetrical cavity of the binding site of F protein inhibitors was located inside the trimer (Figure 3A), at the N-terminal of the F1 or fusion peptide (amino acid residues: Phe137, Phe140, and Leu141) and the beginning of HBR (amino acid residues: Met396, Thr400, Asp486, Glu487, and Phe488). The RV521 reference inhibitor was located symmetrically at the binding site. Thus, the hydrophobic portion of the molecule was located close to the fusion peptide, in the hydrophobic cavity of the site (Figure 3B). The benzimidazole fragment was surrounded by three phenylalanines 488, with which it formed pi–pi stacking interactions (Figure 4A). The protonated -NH3+ group was located in the donor-acceptor region of the site (Figure 3B), forming a salt bridge with Asp486 (Figure 4A). The RMSD value for the redocking procedure was 0.04 Å2, indicating the correctness of the docking protocol. 

#### 2.4.2. Docking Study

The DS-Cav1 binding site was considered a probable site for the binding of (-)-borneol derivatives. Ten docking positions were set as the maximum possible within the framework of the IFD protocol. For some ligands, fewer than 10 positions were implemented (Table S1). Most of the compounds could form hydrogen bonds or salt bridges with the amino acid HRB (Table S1). Salt bridges were formed between the protonated nitrogen atom and the negatively charged amino acids, most often with Asp and Glu. The presence of an aromatic fragment in the compounds (**11a**–**c**, **13-15a**–**c**) was characterized by the formation of pi–pi stacking interactions with Phe of the fusion peptide and/or heptad repeat. In general, a correlation was observed between the molecular docking results and in vitro antiviral activity (Figure S3). Compounds that effectively inhibited RSV were characterized by increased affinity for the binding site. Conversely, inactive compounds showed worse binding affinity to the binding site. 

The analysis of the binding mode of lead compounds **3b** and **5a** in the DS-Cav1 active site revealed that the arrangement was similar to that of the reference inhibitor RV521. The hydrophobic part of compound 3b was located in the hydrophobic cavity near the N-terminus of the F_1_ subunit (Figure 3C). The positively charged nitrogen of compound **3b** was located in the donor-acceptor region, next to the negatively charged amino acid residues Glu487 and Asp486. The hydrophobic 1,7,7-trimethylbicyclo [2.2.1] heptane fragment was surrounded by the amino acid residues of Phe488. In contrast to the RV521 reference inhibitor, molecule **3b** was located closer to the HBR. The key interactions were the hydrogen bond with Asp486 and the salt bridge with Glu487. Given that molecule **3b** was smaller, it did not occupy the entire binding site (Figure 4B). An interesting fact is that molecule **3a** with a shorter linker (n = 1) and molecule **3c** with a longer linker (n = 3) were characterized by similar key interactions, but worse energy parameters (Table S1). We attribute this to the possibility of protonation. For example, deprotonated molecule **3a** was characterized only by hydrophobic interactions with hydrophobic phenylalanine at the binding site (Figure S1), which most likely affects the energy parameters of binding (Table S1). At the same time, protonated compound **3a** formed additional symmetrical salt bridges with Asp486 A and B chains. 

A similar situation was observed for compounds **5a**–**c**. Compound **5b** protonated more easily (pKa = 7.91) than compounds **5a** (pKa = 7.05) and **5c** (pKa = 6.21) (Table S1). Compounds **5a**–**c** were characterized by low inhibitory IC_50_ values. However, the low toxicity of **5a** allowed this compound to be considered as a lead compound. The location in the binding site of deprotonated and protonated forms of compound **5a** was characterized primarily by the formation of hydrophobic interactions. In the case of the deprotonated form, the formation of hydrogen bridges between the oxygen atom of the morpholine fragment and the amino acid residues Phe137 and Arg339 of one of the protomers (Figure S2A) was observed. The binding site arrangement of the protonated form **5a** was similar to that of **3b** (Figure 4C): the hydrophobic 1,7,7-trimethylbicyclo [2.2.1] heptane fragment was surrounded by Phe488 residues of each protomer, with the protonated nitrogen atom of the morpholine fragment forming a hydrogen bond with Asp486 and the Asp486 salt bridge of the adjacent protomer (Figure S2B). Carbonyl oxygen formed hydrogen bridges with Phe488 and Asp489 (Figure 4C). 

#### 2.4.3. Molecular Dynamics (MD) Simulation of Compounds **3b** and **5a**

**Figure 5:**
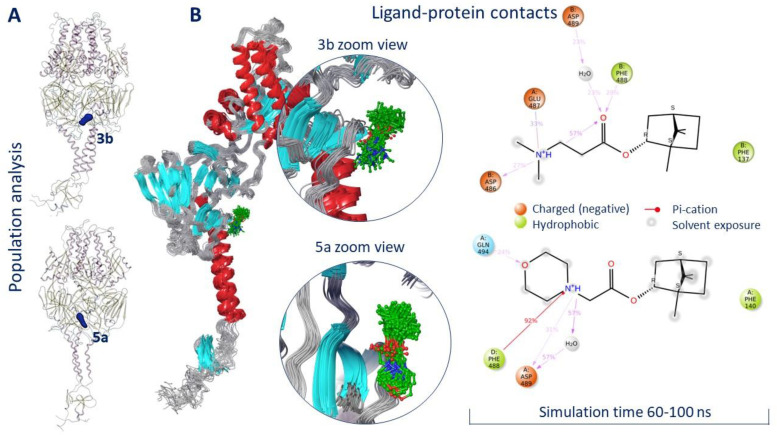
Population analysis (**A**) and ligand–protein contacts (**B**) as result of molecular dynamics simulation.

In order to evaluate the location of ligands **3b** and **5a** within the active binding site, MD simulation was conducted. The model system was created in such a way that the transmembrane domain (amino acids 525–550) of the F protein was immersed in the viral membrane (Figure S4). We believe that in MD simulations of such proteins, the presence of a membrane is necessary for the correct interpretation of the secondary structure of the protein during the entire simulation time. The root mean square deviation (RMSD) was taken as an important basis to measure the stability of the system. According to the RMSD analysis, both systems equalized to 60 ns of simulation (Figures S5 and S6). Ligands **3b** and **5a** were located inside a symmetrical binding site throughout the simulation time. The result of the population analysis indicates minor movements of ligands within the binding site (Figure 5A).

Figure 5B shows an analysis of the location of ligands **3b** and **5a** in the binding site starting from 60 ns of simulation. In both cases, the hydrophobic part of the molecules was located close to the fusion peptide, next to Phe137 in the case of **3b** and next to Phe140 in the case of **5a**. The positively charged parts of the molecule formed hydrogen bridges with negatively charged amino acids. Similar hydrogen bonds with Asp486 and Glu487 were observed for the binding of JNJ-240868 [23] and TMC-353121 inhibitors [17] containing a protonated amino group and a protonated morpholine fragment, respectively. Moreover, it was reported that the mechanism of inhibitory action of known inhibitors of the F protein might be due to the effect of ligands on the conformation of amino acids Phe488, Asp489, and Thr400 [17]. Compounds **3b** and **5a** formed intermolecular interactions with at least two of these amino acids (Figure 5B).

Thus, the results of molecular docking and MD simulations indicate the following: (1) the probable binding site of (-)-borneol derivatives is a symmetrical hydrophobic cavity located inside the F protein between the fusion peptide and the heptad repeat B; (2) lead compounds **3b** and **5a** form strong intermolecular interactions with functional amino acids that persist for a long period of simulation. At the same time, despite the significant structural differences between the compounds being studied and known inhibitors, similar intermolecular interactions (hydrogen and salt bridges, pi–cation stacking bridges) with key amino acids have been recorded.

## 3. Materials and Methods

### 3.1. Chemistry

Reagents and solvents were purchased from commercial suppliers and used as received. Reactions were monitored by gas chromatography-mass spectrometry (GC-MS), using a 7820A gas chromatograph (Agilent Technologies, Santa Clara, CA, USA); flame-ionization detector; HP-5 capillary column (0.25 mm, L 30 m, 0.25 μm), with He as carrier gas (flow rate 2 mL/min, flow division 99:1). ^1^H and ^13^C NMR spectra were recorded on Bruker spectrometers AV-300 (Bruker BioSpin GmbH, Ettlingen, Germany) at 300.13 MHz (^1^H) and 75.47 MHz (^13^C); AV-400 at 400.13 MHz (^1^H) and 100.61 MHz (^13^C); and DRX-500 at 500.13 MHz (^1^H) and 125.76 MHz (^13^C). Chemical shifts (δ values are given in parts per million (ppm) relative to CDCl_3_ [d(CHCl_3_) 7.24, d(CDCl_3_) 76.90 ppm] and coupling constants (J) were measured in Hertz. Splitting patterns were designated as follows: s, singlet; d, doublet; t, triplet; m, multiplet. High-resolution mass spectra (HR-MS) were recorded on a DFS Thermo Scientific spectrometer (Agilent Tech., Santa Clara, CA, USA) in full scan mode (0–500 *m*/*z*, 70 eV electron impact ionization, and direct sample administration). The purity of the target compounds was determined by a gas chromatography method. All of the target compounds reported in this paper had a purity of ≥95%. Compounds **2**–**11a**–**c** were obtained according to previously described methods [12].

### 3.2. General Procedure for the Synthesis of Compounds ***12**–**15a**–**c***

The general procedure for the synthesis of compounds **12a**–**15a** was as follows.

(1S,2R,4S)-1,7,7-Trimethylbicyclo [2.2.1]heptan-2-yl 2-chloroacetate **2a** (1 equiv) was dissolved in CH_3_CN and the appropriate amine (1.2 equiv), K_2_CO_3_ (4 equiv) and KI (1 equiv) were added. The reaction mixture was stirred at room temperature for 12 h. After the reaction was completed, the organic solvent was evaporated. The residue was diluted with brine, extracted twice with CH_2_Cl_2_, dried over anhydrous sodium sulfate, and evaporated to dryness. The obtained product was purified via silica gel column chromatography (hexane-ethyl acetate eluent).

The general procedure for the synthesis of compounds **12**–**15b**–**c** was as follows.

(1S,2R,4S)-1,7,7-Trimethylbicyclo [2.2.1]heptan-2-yl 3-chloropropanoate **2b** or (1S,2R,4S)-1,7,7-trimethylbicyclo [2.2.1]heptan-2-yl 4-chlorobutanoate **2c** (1 equiv) were dissolved in CH_3_CN. Then, the appropriate amine (1.2 equiv), K_2_CO_3_ (4 equiv), and KI (1 equiv) were added. The reaction mixture was stirred at slight heating for 12–48 h. After the reaction was completed, the organic solvent evaporated under reduced pressure. The residue was diluted with brine, extracted twice with CH_2_Cl_2_, dried over anhydrous sodium sulfate, and evaporated to dryness. The obtained product was purified via silica gel column chromatography (hexane-ethyl acetate eluent).

(1S,2R,4S)-1,7,7-Trimethylbicyclo [2.2.1]heptan-2-yl 2-thiomorpholinoacetate (**12a**)

Yellow oil (55% yield, 165 mg); ^1^H NMR (300 MHz, CDCl_3_) δ (ppm): 0.80 (3H, s, Me-10), 0.85 (3H, s, Me-8), 0.88 (3H, s, Me-9), 0.95 (1H, dd, ^2^J = 13.7, J_2endo, 1exo_ = 3.7, H-2endo), 1.15–1.35 (2H, m, H-4endo, H-5exo), 1.62–1.79 (2H, m, H-3, H-4exo), 1.83–1.95 (1H, m, H-5endo), 2.34 (1H, m, H-2exo), 2.65–2.72 (4H, m, 2H-15, 2H-16), 2.80–2.87 (4H, m, 2H-13, 2H-14), 3.23 (2H, s, H-12), 4.91 (1H, m, H-1exo); ^13^C NMR (100 MHz, CDCl_3_) δ (ppm): 170.3 (C-11, quaternary), 79.9 (C-1, CH), 59.9 (C-12, CH_2_), 54.1 (C-13, C-14, CH_2_), 48.3 (C-6, quaternary), 47.3 (C-7, quaternary), 44.4 (C-3, CH), 36.4 (C-2, CH_2_), 27.6 (C-4, CH_2_), 27.5 (C-15, C-16, CH_2_), 26.7 (C-5, CH_2_), 19.2 (Me-9), 18.4 (Me-8), 13.1 (Me-10). IR (ν, cm^−1^): 2954, 2879, 2814, 1745, 1728, 1473, 1454, 1417, 1390, 1365, 1321, 1292, 1275, 1250, 1185, 1161, 1145, 1115, 1082, 1024, 995, 980, 960, 916, 885, 827, 806, 750, 791, 666. HR-MS: 297.1755 (M^+^, C_16_H_27_O_2_N_1_S_1_; calcd 297.1757).

(1S,2R,4S)-1,7,7-trimethylbicyclo [2.2.1]heptan-2-yl 3-thiomorpholinopropanoate (**12b**)

Pale yellow oil (42% yield, 138 mg); ^1^H NMR (300 MHz, CDCl_3_) δ (ppm): 0.81 (3H, s, Me-10), 0.84 (3H, s, Me-8), 0.88 (3H, s, Me-9), 0.95 (1H, dd, ^2^J = 13.7, J_2endo, 1exo_ = 3.7, H-2endo), 1.14–1.34 (2H, m, H-4endo, H-5exo), 1.62–1.78 (2H, m, H-3, H-4exo), 1.84–1.94 (1H, m, H-5endo), 2.25–2.37 (1H, m, H-2exo), 2.46 (2H, t, J = 7.1 Hz, 2H-12), 2.60–2.66 (4H, m, 2H-16, 2H-17), 2.67–2.74 (6H, m, 2H-13, 2H-14, 2H-15), 4.87 (1H, m, H-1exo). ^13^C NMR (100 MHz, CDCl_3_) δ (ppm): 172.6 (C-11, quaternary), 79.7 (C-1, CH), 54.5 (C-14, C-15, CH_2_), 54.4 (C-13, CH_2_), 48.7 (C-6, quaternary), 47.7 (C-7, quaternary), 44.7 (C-3, CH), 36.5 (C-2, CH_2_), 32.4 (C-12, CH_2_), 27.9 (C-4, CH_2_), 27.8 (C-16, C-17, CH_2_), 27.0 (C-5, CH_2_), 19.5 (Me-9), 18.7 (Me-8), 13.4 (Me-10). IR (ν, cm^−1^): 2953, 2877, 2810, 2775, 1732, 1668, 1456, 1417, 1377, 1345, 1321, 1302, 1275, 1252, 1228, 1181, 1159, 1115, 1059, 1024, 997, 980, 958, 914, 887, 825, 777, 752. HR-MS: 311.1912 (M^+^, C_17_H_29_O_2_N_1_^32^S_1_; calcd 311.1914).

(1S,2R,4S)-1,7,7-trimethylbicyclo [2.2.1]heptan-2-yl 4-thiomorpholinobutanoate (**12c**)

Yellow oil (37% yield, 111 mg); ^1^H NMR (400 MHz, CDCl_3_) δ (ppm): 0.79 (3H, s, Me-10), 0.84 (3H, s, Me-8), 0.87 (3H, s, Me-9), 0.92 (1H, dd, ^2^J = 13.8, J_2endo, 1exo_ = 3.3, H-2endo), 1.14–1.31 (2H, m, H-4endo, H-5exo), 1.62–1.81 (4H, m, H-3, H-4exo, 2H-13), 1.85–1.93 (1H, m, H-5endo), 2.27–2.38 (5H, m, H-2exo, 2H-12, 2H-14), 2.61–2.71 (8H, m, 2H-15, 2H-16, 2H-17, 2H-18), 4.84 (1H, m, H-1exo). ^13^C NMR (125 MHz, CDCl_3_) δ (ppm):172.6 (C-11, quaternary), 79.7 (C-1, CH), 54.5 (C-14, C-15, CH_2_), 54.4 (C-13, CH_2_), 48.7 (C-6, quaternary), 47.7 (C-7, quaternary), 44.7 (C-3, CH), 36.5 (C-2, CH_2_), 32.4 (C-12, CH_2_), 27.9 (C-4, CH_2_), 27.8 (C-16, C-17, CH_2_), 27.0 (C-5, CH_2_), 19.5 (Me-9), 18.7 (Me-8), 13.4 (Me-10). IR (ν, cm^−1^): 2953, 2878, 2809, 2771, 1731, 1684, 1453, 1417, 1375, 1346, 1316, 1304, 1280, 1255, 1232, 1205, 1177, 1157, 1115, 1078, 1023, 995, 980, 957, 914, 887, 823, 774, 754. HR-MS: 325.2072 (M^+^, C_18_H_31_O_2_N_1_^32^S_1_; calcd 325.2070).

(1S,2R,4S)-1,7,7-trimethylbicyclo [2.2.1]heptan-2-yl 2-(4-benzylpiperidin-1-yl)acetate (**13a**)

Yellow oil (51% yield, 357 mg); ^1^H NMR (400 MHz, CDCl_3_) δ (ppm): 0.79 (3H, s, Me-10), 0.84 (3H, s, Me-8), 0.87 (3H, s, Me-9), 0.94 (1H, dd, ^2^J = 13.9, J_2endo, 1exo_ = 3.4, H-2endo), 1.15–1.41 (4H, m, H-4endo, H-5exo, H-15a, H-16a), 1.43–1.54 (1H, m, H-17), 1.57–1.76 (4H, m, H-3, H-4exo, H-15e, H-16e), 1.84–1.94 (1H, m, H-5endo), 2.06–2.15 (2H, m, H-13a, H-14a), 2.29–2.38 (1H, m, H-2exo), 2.51 (2H, d, J = 6.9 Hz, H-18), 2.87–2.94 (2H, br. m., H-13e, H-14e), 3.19 (2H, s, H-12), 4.87–4.93 (1H, m, H-1exo), 7.09–7.14 (2H, m, H-20, H-21), 7.15–7.18 (1H, m, H-24), 7.22–7.28 (2H, m, H-22, H-23). ^13^C NMR (125 MHz, CDCl_3_) δ (ppm): 170.8 (C-11, quaternary), 140.5 (C-18, quaternary), 128.9 (C-22, C-23, CH), 128.0 (C-20, C-21, CH), 125.6 (C-24, CH), 79.9 (C-1, CH), 59.7 (C-12, CH_2_), 53.3 (C-13, C-14, CH_2_), 48.6 (C-6, quaternary), 47.6 (C-7, quaternary), 44.6 (C-3, CH), 43.0 (C-18, CH_2_), 37.3 (C-17, CH), 36.6 (C-2, CH_2_), 31.9 (C-15, C-16, CH_2_), 27.8 (C-4, CH_2_), 26.9 (C-5, CH_2_), 19.5 (Me-9), 18.6 (Me-8), 13.4 (Me-10). HR-MS: 369.2668 (M^+^, C_24_H_35_O_2_N_1_; calcd 369.2662).

(1S,2R,4S)-1,7,7-trimethylbicyclo [2.2.1]heptan-2-yl 3-(4-benzylpiperidin-1-yl)propanoate (**13b**)

Pale yellow oil (47% yield, 164 mg); ^1^H NMR (400 MHz, CDCl_3_) δ (ppm): 0.80 (3H, s, Me-10), 0.85 (3H, s, Me-8), 0.87 (3H, s, Me-9), 0.95 (1H, dd, ^2^J = 13.6, J_2endo, 1exo_ = 3.6, H-2endo), 1.14–1.30 (4H, m, H-4endo, H-5exo, H-16a, H-17a), 1.43–1.55 (1H, m, H-18), 1.57–1.77 (4H, m, H-3, H-4exo, H-16e, H-17e), 1.85–1.95 (3H, m, H-5endo, H-14a, H-15a), 2.26–2.36 (1H, m, H-2exo), 2.44–2.53 (4H, m, H-19, H-12), 2.61–2.67 (2H, m, H-13), 2.82–2.89 (2H, br. m., H-14e, H-15e), 4.84–4.89 (1H, m, H-1exo), 7.09–7.14 (2H, m, H-21, H-22), 7.15–7.18 (1H, m, H-25), 7.22–7.28 (2H, m, H-23, H-24). ^13^C NMR (125 MHz, CDCl_3_) δ (ppm): 172.9 (C-11, quaternary), 140.5 (C-19, quaternary), 128.9 (C-23, C-24, CH), 128.0 (C-21, C-22, CH), 125.6 (C-25, CH), 79.6 (C-1, CH), 53.9 (C-13, CH_2_), 53.5 and 53.4 (C-14, C-15, CH_2_), 48.6 (C-6, quaternary), 47.6 (C-7, quaternary), 44.7 (C-3, CH), 43.1 (C-19, CH_2_), 37.7 (C-18, CH), 36.5 (C-2, CH_2_), 32.8 (C-12, CH_2_), 32.1 and 32.0 (C-16, C-17, CH_2_), 27.9 (C-4, CH_2_), 26.9 (C-5, CH_2_), 19.5 (Me-9), 18.7 (Me-8), 13.3 (Me-10). IR (ν, cm^−1^): 3084, 3062, 3026, 2950, 2883, 2848, 2806, 2773, 1733, 1666, 1604, 1494, 1467, 1453, 1377, 1348, 1302, 1251, 1234, 1194, 1180, 1159, 1132, 1113, 1055, 1029, 997, 977, 887, 823, 746, 700, 591, 505. HR-MS: 383.2824 (M^+^, C_25_H_37_O_2_N_1_; calcd 383.2819).

(1S,2R,4S)-1,7,7-trimethylbicyclo [2.2.1]heptan-2-yl 4-(4-benzylpiperidin-1-yl)butanoate (**13c**)

Yellow oil (32% yield, 96 mg); ^1^H NMR (400 MHz, CDCl_3_) δ (ppm): 0.79 (3H, s, Me-10), 0.84 (3H, s, Me-8), 0.87 (3H, s, Me-9), 0.92 (1H, dd, ^2^J = 13.8, J_2endo, 1exo_ = 3.4, H-2endo), 1.15–1.32 (4H, m, H-4endo, H-5exo, H-16a, H-17a), 1.42–1.53 (1H, m, H-19), 1.56–1.95 (10H, m, H-3, H-4exo, H-5endo, H-17e, H-18e, H-17a, H-18a, H-13, H-15a, H-16a), 2.25–2.36 (1H, m, H-2exo, H-12, H-14), 2.50 (2H, d, J = 7.0 Hz, H-20), 2.83–2.89 (2H, br. m., H-15e, H-16e), 4.83–4.88 (1H, m, H-1exo), 7.08–7.14 (2H, m, H-22, H-23), 7.15–7.18 (1H, m, H-26), 7.22–7.27 (2H, m, H-24, H-25). ^13^C NMR (125 MHz, CDCl_3_) δ (ppm): 173.7 (C-11, quaternary), 140.5 (C-20, quaternary), 128.9 (C-24, C-25, CH), 127.9 (C-22, C-23, CH), 125.6 (C-26, CH), 79.5 (C-1, CH), 58.0 (C-14, CH_2_), 53.8 and 53.7 (C-15, C-16, CH_2_), 48.5 (C-6, quaternary), 47.6 (C-7, quaternary), 44.6 (C-3, CH), 43.1 (C-20, CH_2_), 37.8 (C-19, CH), 36.6 (C-2, CH_2_), 32.6 (C-12, CH_2_), 32.0 (C-16, C-17, CH_2_), 27.9 (C-4, CH_2_), 26.9 (C-5, CH_2_), 22.3 (C-13, CH_2_), 19.5 (Me-9), 18.6 (Me-8), 13.4 (Me-10). HR-MS: 397.2972 (M^+^, C_25_H_37_O_2_N_1_; calcd 397.2975).

(1S,2R,4S)-1,7,7-Trimethylbicyclo [2.2.1]heptan-2-yl 2-(4-phenylpiperazin-1-yl)acetate (**14a**)

Yellow oil (88% yield, 440 mg); ^1^H NMR (400 MHz, CDCl_3_) δ (ppm): 0.82 (3H, s, Me-10), 0.86 (3H, s, Me-9), 0.89 (3H, s, Me-8), 0.98 (1H, dd, ^2^*J* = 13.8, J_2endo, 1exo_ = 3.4, H-2endo), 1.17–1.34 (2H, m, H-4endo, H-5exo), 1.64–1.67 (1H, m, H-3), 1.69–1.79 (1H, m, H-4exo), 1.86–1.94 (1H, m, H-5endo), 2.32–2.41 (1H, m, H-2exo), 2.75 (4H, t, *J* = 5.0, 2H-13, 2H-16), 3.24 (4H, t, *J* = 5.0, 2H-14, 2H-15), 3.28 (2H, s, 2H-12), 4.94 (1H, m, H-1exo), 6.81–6.86 (1H, m, H-20), 6.89–6.94 (2H, m, H-18, H-22), 7.21–7.27 (2H, m, H-19, H-21). ^13^C NMR (100 MHz, CDCl_3_) δ (ppm): 170.3 (C-11, quaternary), 151.1 (C-17, quaternary), 128.9 (C-19, C-21, CH), 119.6 (C-20, CH), 115.9 (C-18, C-22, CH), 80.2 (C-1, CH), 59.3 (C-12, CH_2_), 52.8 (C-13, C-16, CH_2_), 48.9 (C-14, C-15, CH_2_), 48.6 (C-6, quaternary), 47.7 (C-7, quaternary), 44.7 (C-3, CH), 36.7 (C-2, CH_2_), 27.9 (C-4, CH_2_), 26.9 (C-5, CH_2_), 19.6 (Me-9), 18.7 (Me-8), 13.4 (Me-10). IR (ν, cm^−1^): 3091, 3061, 3037, 3024, 2953, 2879, 2821, 2762, 1746, 1682, 1600, 1579, 1502, 1452, 1423, 1387, 1336, 1304, 1288, 1265, 1232, 1200, 1178, 1158, 1115, 1080, 1024, 993, 982, 952, 928, 883, 833, 758, 692, 525. HR-MS: 356.2454 (M^+^, C_22_H_32_O_2_N_2_; calcd 356.2458).

(1S,2R,4S)-1,7,7-Trimethylbicyclo [2.2.1]heptan-2-yl 3-(4-phenylpiperazin-1-yl)propanoate (**14b**)

Pale yellow solid (91% yield, 364 mg); mp 83.0 °C; ^1^H NMR (400 MHz, CDCl_3_) δ (ppm): 0.82 (3H, s, Me-10), 0.85 (3H, s, Me-9), 0.88 (3H, s, Me-8), 0.98 (1H, dd, ^2^*J* = 13.8, J_2endo, 1exo_ = 3.4, H-2endo), 1.16–1.33 (2H, m, H-4endo, H-5exo), 1.65–1.67 (1H, m, H-3), 1.68–1.78 (1H, m, H-4exo), 1.88–1.96 (1H, m, H-5endo), 2.28–2.37 (1H, m, H-2exo), 2.53 (2H, t, *J* = 7.1, H-12), 2.62 (4H, t, *J* = 5.0, 2H-14, 2H-17), 2.74 (2H, t, *J* = 7.2, H-13), 3.17 (4H, t, *J* = 4.9, 2H-15, 2H-16), 4.90 (1H, m, H-1exo), 6.81–6.85 (1H, m, H-21), 6.88–6.92 (2H, m, H-19, H-23), 7.21-7.27 (2H, m, H-20, H-22). ^13^C NMR (100 MHz, CDCl_3_) δ (ppm): 172.6 (C-11, quaternary), 151.2 (C-18, quaternary), 128.9 (C-20, C-22, CH), 119.6 (C-21, CH), 115.9 (C-19, C-23, CH), 79.8 (C-1, CH), 53.7 (C-13, CH_2_), 52.8 (C-14, C-17, CH_2_), 48.9 (C-15, C-16, CH_2_), 48.7 (C-6, quaternary), 47.7 (C-7, quaternary), 44.7 (C-3, CH), 36.6 (C-2, CH_2_), 32.7 (C-12, CH_2_), 27.9 (C-4, CH_2_), 27.0 (C-5, CH_2_), 19.6 (Me-9), 18.7 (Me-8), 13.4 (Me-10). IR (ν, cm^−1^): 3091, 3059, 3026, 2953, 2880, 2831, 2794, 1723, 1600, 1577, 1498, 1450, 1433, 1406, 1378, 1363, 1342, 1317, 1263, 1240, 1199, 1171, 1146, 1113, 1078, 1017, 993, 926, 881, 827, 759, 694, 524. HR-MS: 370.2612 (M^+^, C_23_H_34_O_2_N_2_; calcd 370.2615).

(1S,2R,4S)-1,7,7-trimethylbicyclo [2.2.1]heptan-2-yl 4-(4-phenylpiperazin-1-yl)butanoate (**14c**)

Yellow oil (88% yield, 352 mg); ^1^H NMR (400 MHz, CDCl_3_) δ (ppm): 0.81 (3H, s, Me-10), 0.85 (3H, s, Me-9), 0.88 (3H, s, Me-8), 0.94 (1H, dd, ^2^*J* = 13.7, J_2endo, 1exo_ = 3.5, H-2endo), 1.16–1.32 (2H, m, H-4endo, H-5exo), 1.65–1.67 (1H, m, H-3), 1.68–1.77 (1H, m, H-4exo), 1.85 (2H, quintet, *J* = 7.4, H-13), 1.89–1.96 (1H, m, H-5endo), 2.29–2.34 (1H, m, H-2exo), 2.37 (2H, t, *J* = 7.5, H-12), 2.41 (2H, t, *J* = 7.5, H-14), 2.59 (4H, t, *J* = 5.0, 2H-15, 2H-18), 3.18 (4H, t, *J* = 5.0, 2H-16, 2H-17), 4.87 (1H, m, H-1exo), 6.80–6.85 (1H, m, H-22), 6.89–6.92 (2H, m, H-20, H-24), 7.21–7.26 (2H, m, H-21, H-23). ^13^C NMR (100 MHz, CDCl_3_) δ (ppm): 173.6 (C-11, quaternary), 151.1 (C-19, quaternary), 128.9 (C-21, C-23, CH), 119.5 (C-22, CH), 115.8 (C-20, C-24, CH), 79.6 (C-1, CH), 57.5 (C-14, CH_2_), 53.0 (C-15, C-18, CH_2_), 48.9 (C-16, C-17, CH_2_), 48.6 (C-6, quaternary), 47.7 (C-7, quaternary), 44.7 (C-3, CH), 36.7 (C-2, CH_2_), 32.4 (C-12, CH_2_), 27.9 (C-4, CH_2_), 26.9 (C-5, CH_2_), 22.1 (C-13), 19.5 (Me-9), 18.7 (Me-8), 13.4 (Me-10). IR (ν, cm^−1^): 3092, 3061, 3038, 3024, 2953, 2879, 2818, 2777, 1731, 1600, 1580, 1501, 1454, 1419, 1379, 1356, 1302, 1234, 1180, 1159, 1139, 1113, 1076, 1023, 993, 980, 962, 926, 876, 824, 758, 692, 524. HR-MS: 384.2773 (M^+^, C_23_H_34_O_2_N_2_; calcd 384.2771.

(1S,2R,4S)-1,7,7-trimethylbicyclo [2.2.1]heptan-2-yl 2-(4-(2-fluorophenyl)piperazin-1-yl)acetate (**15a**)

Yellow oil (53% yield, 265 mg); ^1^H NMR (300 MHz, CDCl_3_) δ (ppm): 0.82 (3H, s, Me-10), 0.85 (3H, s, Me-8), 0.89 (3H, s, Me-9), 0.98 (1H, dd, ^2^J = 14.2, J_2endo, 1exo_ = 3.5, H-2endo), 1.16–1.34 (2H, m, H-4endo, H-5exo), 1.64–1.79 (2H, m, H-3, H-4exo), 1.84–1.95 (1H, m, H-5endo), 2.30–2.42 (2H, m, H-2exo), 2.51 (2H, d, J = 6.9 Hz, H-18), 2.71–2.82 (4H, m, H-13, H-14), 3.09–3.20 (4H, m, H-15, H-16), 3.28 (2H, s, H-12), 4.89–5.00 (1H, m, H-1exo), 6.85–7.07 (4H, m, H-18, H-19, H-20, H-21, H-22). ^13^C NMR (125 MHz, CDCl_3_) δ (ppm): 170.5 (C-11, quaternary), 155.5 (d J = 249.6 Hz, C-22, CH), 139.9 (d J = 8.8 Hz, C-17, CH), 124.3 (d J = 3.6 Hz, C-19, CH), 122.3 (d J = 7.9 Hz, C-20, CH), 118.8 (d J = 2.9 Hz, C-18, CH), 115.9 (d, J = 25.5 Hz, C-21, CH), 80.2 (C-1, CH), 59.4 (C-12, CH_2_), 52.9 (C-13, C-14, CH_2_), 50.2 (C-15, C-16, CH_2_), 48.6 (C-6, quaternary), 47.7 (C-7, quaternary), 44.6 (C-3, CH), 36.6 (C-2, CH_2_), 27.9 (C-4, CH_2_), 26.9 (C-5, CH_2_), 19.6 (Me-9), 18.7 (Me-8), 13.5 (Me-10). IR (ν, cm^−1^): 3066, 3037, 2953, 2881, 2825, 2760, 2706, 1745, 1682, 1612, 1579, 1502, 1454, 1389, 1342, 1304, 1290, 1238, 1203, 1174, 1159, 1140, 1113, 1082, 1025, 993, 982, 931, 885, 829, 810, 753, 731, 470. HR-MS: 374.2357 (M^+^, C_22_H_31_O_2_N_2_F; calcd 374.2364).

(1S,2R,4S)-1,7,7-trimethylbicyclo [2.2.1]heptan-2-yl 3-(4-(2-fluorophenyl)piperazin-1-yl)propanoate (**15b**)

Pale yellow solid (42% yield, 168 mg); ^1^H NMR (300 MHz, CDCl_3_) δ (ppm): 0.82 (3H, s, Me-10), 0.85 (3H, s, Me-8), 0.88 (3H, s, Me-9), 0.98 (1H, dd, ^2^J = 14.4, J_2endo, 1exo_ = 3.4, H-2endo), 1.16–1.34 (2H, m, H-4endo, H-5exo), 1.62–1.78 (2H, m, H-3, H-4exo), 1.86–1.97 (1H, m, H-5endo), 2.28–2.38 (2H, m, H-2exo), 2.48–2.78 (9H, m, H-13, H-12, H-14, H-15), 3.02-3.15 (4H, m, H-16, H-17), 4.85–4.95 (1H, m, H-1exo), 6.86–7.06 (4H, m, H-19, H-21, H-22, H-23). ^13^C NMR (75 MHz, CDCl_3_) δ (ppm): 172.6 (C-11, quaternary), 155.5 (d J = 246.2 Hz, C-20, CH), 140.0 (d J = 9.0 Hz, C-18, CH), 124.3 (d J = 3.6 Hz, C-21, CH), 122.3 (d J = 7.5 Hz, C-23, CH), 118.7 (d J = 3.0 Hz, C-19, CH), 115.9 (d, J = 24.9 Hz, C-22, CH), 79.7 (C-1, CH), 53.6 (C-13, CH_2_), 52.8 (C-14, C-15, CH_2_), 50.4 (C-16, C-17, CH_2_), 48.7 (C-6, quaternary), 47.7 (C-7, quaternary), 44.7 (C-3, CH), 36.5 (C-2, CH_2_), 32.7 (C-12, CH_2_), 27.9 (C-4, CH_2_), 27.0 (C-5, CH_2_), 19.6 (Me-9), 18.7 (Me-8), 13.4 (Me-10). IR (ν, cm^−1^): 3066, 3037, 2982, 2953, 2879, 2824, 2783, 2694, 1731, 1655, 1612, 1578, 1501, 1454, 1377, 1356, 1336, 1302, 1282, 1261, 1239, 1207, 1194, 1161, 1142, 1113, 1068, 1051, 1038, 1026, 1016, 999, 982, 957, 937, 887, 825, 812, 777, 757, 737, 559, 470. HR-MS: 388.2522 (M^+^, C_23_H_33_O_2_N_2_F; calcd 388.2521).

(1S,2R,4S)-1,7,7-trimethylbicyclo [2.2.1]heptan-2-yl 4-(4-(2-fluorophenyl)piperazin-1-yl)butanoate (**15c**)

Pale yellow solid (47% yield, 235 mg); ^1^H NMR (300 MHz, CDCl_3_) δ (ppm): 0.81 (3H, s, Me-10), 0.85 (3H, s, Me-8), 0.88 (3H, s, Me-9), 0.94 (1H, dd, ^2^J = 13.6, J_2endo, 1exo_ = 3.4, H-2endo), 1.15–1.33 (2H, m, H-4endo, H-5exo), 1.62–1.96 (5H, m, H-3, H-4exo, H-5endo, H-13), 2.27–2.46 (5H, m, H-2exo, H-12, H-14), 2.57–2.67 (4H, m, H-15, H-16), 3.05–3.13 (4H, m, H-17, H-18), 4.82–4.93 (1H, m, H-1exo), 6.85–7.09 (4H, m, H-20, H-21, H-22, H-23). ^13^C NMR (75 MHz, CDCl_3_) δ (ppm): 173.7 (C-11, quaternary), 155.7 (d J = 241.3 Hz, C-24, CH), 140.1 (d J = 8.8 Hz, C-19, CH), 124.3 (d J = 3.9 Hz, C-21, CH), 122.3 (d J = 7.9 Hz, C-22, CH), 118.8 (d J = 3.2 Hz, C-20, CH), 115.9 (d, J = 23.3 Hz, C-23, CH), 79.6 (C-1, CH), 57.6 (C-14, CH_2_), 53.1 (C-15, C-16, CH_2_), 50.4 (C-17, C-18, CH_2_), 48.6 (C-6, quaternary), 47.6 (C-7, quaternary), 44.7 (C-3, CH), 36.7 (C-2, CH_2_), 32.4 (C-12, CH_2_), 27.9 (C-4, CH_2_), 27.0 (C-5, CH_2_), 22.2 (C-13, CH_2_), 19.6 (Me-9), 18.7 (Me-8), 13.4 (Me-10). IR (ν, cm^−1^): 3063, 3037, 2952, 2877, 2825, 2771, 2686, 1735, 1680, 1612, 1576, 1502, 1466, 1452, 1412, 1377, 1356, 1317, 1302, 1282, 1271, 1255, 1238, 1209, 1174, 1159, 1147, 1113, 1074, 1059, 1037, 1020, 1007, 995, 982, 955, 931, 887, 866, 814, 804, 749, 725, 468. HR-MS: 402.2678 (M^+^, C_24_H_35_O_2_N_2_F; calcd 402.2677).

### 3.3. MTT Cytotoxicity Assay

The in vitro cytotoxicity of the studied compounds was determined using the MTT assay with non-infected cells. The compounds were weighed in an amount of 2 mg and dissolved in 100 μL of DMSO. Then, the resulting solution was adjusted with the medium to a concentration of 1000 μg/mL, and a series of 2-fold dilutions was prepared. The final concentration of DMSO was more than twice lower than its 50% cytotoxic con-centration, and thus, could not affect cell viability. A one-day culture of HEp2 cells, grown in 96-well plates, with cell concentration 3 × 10^5^/well of the plate, was checked visually in an inverted microscope for the integrity of the monolayer. Plates were selected for work, where the cell closure was 60–80%. Dilutions of the compounds at the appropriate concentration were added to the plate in a volume of 100 μL in each well in 2 replicates for each tested concentration. The plates were incubated for 24 h at 37 °C in the presence of 5% CO_2_. Cell viability was assessed using the MTT test. The MTT solution was prepared on maintenance medium at a concentration of 0.5 mg/mL. Then, 0.1 mL of MTT solution was added to each well. After 1.5 h of MTT contact at 37 °C at a CO_2_ concentration of 5%, MTT was discarded with the cells of the well, and 0.1 mL of 96% ethyl alcohol was poured, after which the optical density in the wells was measured at 535 nm. Based on the data obtained, the CC_50_ was calculated.

### 3.4. Antiviral Assay

The respiratory syncytial virus (strain A2) and cell culture HEp-2 were obtained from the working collection of the Laboratory of Chemotherapy for Viral Infections of Smorodintsev Research Institute of Influenza. The antiviral activity against the respiratory syncytial virus (RSV A strain A2) was assessed in a series of 3-fold dilutions of test compounds, starting from ½CC_50_, which were added to HEp-2 cell culture at a double concentration, 100 μL per well, followed by an addition of 100 μL of the virus in a series of 10-fold dilutions. The cells were incubated at 37 °C and 5% CO_2_ for 1 h. Then, the virus was washed out, and the compounds were again added at a single concentration and incubated at 37 °C and 5% CO_2_ for 6 days. For the enzyme-linked immunosorbent assay (ELISA), the cell culture was fixed with cold 80% acetone at −20 °C for 15 min and then washed with phosphate-buffered saline containing 0.05% Tween 20. Next, a solution of primary mouse anti-RSV F protein antibodies was added to the culture and incubated at room temperature under continuous stirring for 2 h. Then, the cells were again washed with buffer, secondary anti-mouse antibodies were added, and the cells were incubated under continuous stirring for 2 h. Then, the antibodies were washed off, and a substrate-chromogenic mixture with tetramethylbenzidine was added. After 5 min, the reaction was stopped with 0.1 M of sulfuric acid, and the optical density of the solution was measured at a wavelength of 450 nm. Wells with absorbance values of two-fold or greater than the cell control were considered contaminated. The virus titer was calculated using the Reed and Muench method. All experiments were performed in triplicate.

### 3.5. Time-of-Addition Assay

Compounds **3b**, **5a**, and **6b** were added at different time points before, after, or simultaneously with the introduction of the virus. The time of addition of the compound was counted from point 0—the time of the entry of the virus into the cell. During the period (-1)–0, the cells together with the virus were incubated at 40 °C. All other experiments were carried out at 37 °C. RSV virus A (2 mL) was added to the cells at a time that was conventionally designated as point -1, after which the cells were kept for an hour at a temperature of 40 °C. Then, at point 0, the virus was unbound. The cells were transferred to a thermostat at 37 °C, where they were incubated for 25 h. After this period, the medium was taken from each well, and a series of ten-fold dilutions were made on fresh cell culture and incubated for 6 days. For each compound, 2 repetitions were made by different operators. The virus titer was estimated by ELISA. The compounds were added at the following times relative to the addition of the virus: point -2—the compound was introduced one hour before cell infection (prophylactic regimen); point 0—at the moment of temperature change; points 1, 2, 4, 6, 24, at 1, 2, 4, 6, and 24 h after the temperature change, respectively. In the wells marked (-2)–(25), the compound was kept throughout the experiment, starting from point -2 and until the end of the experiment—25 h. No compound was added to the control wells. Instead, a similar volume of medium was added.

### 3.6. Molecular Modeling

#### 3.6.1. Ligands and Protein Preparations

The geometric parameters of the ligands were optimized by the OPLS4 force field method [24], considering possible conformers. To estimate the ADME parameters of the ligands, we used the QikProp plugin. The ADME estimation method is based on the molecular description of structures. Acidity constants for some compounds were calculated using quantum-chemical software Jaguar [25]. The most popular DFT method, B3LYP [26] with Danning’s basis set cc-pVTZ(+) [27], was used for the optimization procedure. The contribution of protonated and deprotonated molecules in the molecular docking procedure was considered on the basis of the Henderson–Hasselbalch equation. The geometrical parameters of the full-size F protein (PDB code 7LVW [28]) of human RVS A2 were downloaded from the noncommercial base data Protein Data Bank [29]. To localize the binding site complex, the protomer-inhibitor RV521 was used (PDB code 7KQD [19]). Model structures were prepared using the plugin Protein Preparation Wizard: hydrogen atoms were added and minimized, side chains of amino acids were edited, multiple chemical bonds were restored, and water molecules were removed. Geometric parameters were optimized in the OPLS4 force field [24].

#### 3.6.2. Molecular Docking

All theoretical calculations were carried out using Schrodinger Small Molecule Drug Discovery Release 2022–1 software. The molecular docking of compounds was carried out using the induced fit docking (IFD) protocol with the following conditions: flexible protein and ligand, grid matrix size of 15 Å, and amino acids within a radius of 5 Å from the ligand were optimized considering the influence of the ligand. The ranking of docking solutions was carried out by evaluating the following calculation parameters: the docking score (based on GlideScore minus fines), the ligand efficiency (LE) and the parameter of the model energy value (Emodel), including the GlideScore value, the energy of unrelated interactions, and the parameters of the energy spent for the formation of the binding site stacking.

#### 3.6.3. Molecular Dynamics

Trimer–ligand complexes for compounds **3b** and **5a** were used for subsequent molecular dynamics simulations. Given the structure of the full-length F protein, the transmembrane domain (525–550 amino acids) was placed in the POPC membrane (phosphatidylcholine). Phosphatidylcholine is a part of most cell membranes of viruses [30,31]. The complexes were placed in an orthorhombic box with a size of 15 × 35 × 55 Å, filled with 0.15 M of an aqueous solution of NaCl. The solvent model was TIP3P. Counterions were added to the system to maintain neutrality. The thermodynamic ensemble was NPγT. The period of recorded dynamics simulation was 100 nanoseconds at a temperature of 310 K (37 °C). The protocol for preparing the system for simulation included a preliminary minimization and the balancing of system components.

The population analysis was performed using the VolMap plug-in implemented in the VMD program [32]. A map based on the weighted atomic density and weighted atomic population was considered. To quantify protein structure changes before and after simulation, we used the MultiSeq plug-in implemented in the VMD program. The RMSD parameter was chosen as a change descriptor. The changes were visually represented as a color gradient (Blue-White-Red).

## 4. Conclusions

A series of thirty-nine esters based on (-)-borneol were synthesized by a simple three-step procedure and evaluated for antiviral activity against RSV. The cytotoxicity of the compounds tested was studied in HEp2 cells. Two of the (-)-borneol derivatives synthesized, **3b** (with methyl substitutions at the nitrogen atom) and **5a** (with morpholine cycle), demonstrated the best antiviral activities. Moreover, the derivatives with piperidine (**6b**), 4-methylpiperidine (**7b**), 4-metylpiperazine (**9c**), and 4-ethylpiperazine (**10b**,**c**) were found to exhibit significant antiviral activity. The time-of-addition experiments demonstrated that compounds **3b**, **5a**, and **6b** were likely to exhibit inhibitory activities against RSV infection by binding to the viral F protein and blocking the fusion of the viral and host cell membranes. Docking studies and molecular dynamics simulation on a representative target (F protein) revealed that the tested compounds fit well into RSV F hydrophobic cavity DS-Cav1. To conclude, (-)-borneol ester with an N-containing heterocycle has been identified as a promising therapeutic candidate for developing a broad-spectrum antiviral agent.

## Figures and Tables

**Table 1:** Structure and antiviral activity of (-)-borneol-based esters.

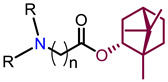					
Virus	n	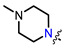	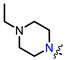	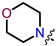	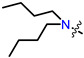
		I	II	III	IV
Filoviruses	1	^a^ SI_MarV-GP_ = 14	^a^ SI_MarV-GP_ = 29	^a^ SI_MarV-GP_ = 5	^a^ SI_MarV-GP_ = 6
	1	^b^ SI_EboV-GP_ = 46	^b^ SI_EboV-GP_ = 80	^b^ SI_EboV-GP_ = 13	NT
	2	^a^ SI_MarV-GP_ = 20	^a^ SI_MarV-GP_ = 47	^a^ SI_MarV-GP_ = 5	^a^ SI_MarV-GP_ = 15
	2	^b^ SI_EboV-GP_ = 870	^b^ SI_EboV-GP_ = 170	^b^ SI_EboV-GP_ = 36	NT
Influenza virus	1	^c^ SI_H1N1_ = 23	^c^ SI_H1N1_ = 16	^c^ SI_H1N1_ = 82	^c^ SI_H1N1_ = 16
	2	^c^ SI_H1N1_ = 25	^c^ SI_H1N1_ = 12	^c^ SI_H1N1_ = 45	^c^ SI_H1N1_ = 12
Vaccinia virus	1	NA	NA	^d^ SI_VACV_ = 56	NA
	2	NA	NA	^d^ SI_VACV_ = 48	NA
	3	NA	NA	^d^ SI_VACV_ = 23	NA
Coronavirus	1	NA	NA	^e^ SI_pSARS-CoV-2-S_ = 5	^e^ SI_pSARS-CoV-2-S_ = 10
					SI_SARS-CoV-2_ = 11–40 *
	2	NA	NA	^e^ SI_pSARS-CoV-2-S_ = 18	^e^ SI_pSARS-CoV-2-S_ = 29
					SI_SARS-CoV-2_ = 6–102 *

The selectivity index (SI, CC_50_/IC_50_ ratio) values of each compound were taken as the evaluation reference for antiviral potency. The antiviral activity against filoviruses and SARS-CoV-2 was studied using a “surrogate” system. This system included vesicular stomatitis virus (VSV) particles pseudotyped with Ebola virus GP (rVSV-ΔG-EboV-GP) or Marburg virus GP (rVSV-ΔG-MarV-GP), and lentiviral particles exhibiting the S protein of SARS-CoV-2 (pSARS-CoV-2-S). ^a^ SI_MarV-GP_ = CC_50_/IC_50MarV-GP_, where IC_50MarV-GP_ is the concentration of a compound required to inhibit rVSV-ΔG-MarV-GP infection of HEK293T cells by 50%. ^b^ SI_EboV-GP_ = CC_50_/IC_50EboV-GP_, where IC_50EboV-GP_ is the concentration of a compound required to inhibit rVSV-ΔG-EboV-GP infection of HEK293T cells by 50%. ^c^ SI_H1N1_ = CC_50_/IC_50H1N1_, where IC_50H1N1_: concentration resulting in a decrease in the A/Puerto Rico/8/34 (H1N1) virus titer by 50%. ^d^ SI_VACV_ = CC_50_/IC_50VACV_, where IC_50VACV_: 50% inhibitory concentration, at which 50% of cells in infected monolayers are preserved. ^e^ SI_pSARS-CoV-2-S_ = CC_50_/IC_50pSARS-CoV-2-S_, where IC_50pSARS-CoV-2-S_ is the concentration of a compound required to inhibit SARS-CoV-2 pseudoviruses by 50%. SI_SARS-CoV-2_ = CC_50_/IC_50SARS-CoV-2_, where IC_50SARS-CoV-2_ is the concentration leading to 50% inhibition of virus replication. * The data are presented against various SARS-CoV-2 virus strains. CC_50_ is the cytotoxic concentration, the concentration resulting in the death of 50% of cells.

**Table 2:** In vitro anti-RSV activity, cytotoxicity, and logP values for compounds **3**–**15a**–**c**.

Compound	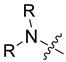	n	logP	CC_50_^a^, µM	IC_50_^b^, µM	SI ^c^
**3a**	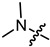	1	2.364	497.2 ± 53.2	133.7 ± 19.5	4
**3b**	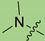	2	2.733	982.7 ± 44.2	8.9 ± 2.4	111
**3c**	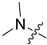	3	3.241	228.1 ± 23.5	23.9 ± 3.7	10
**4a**	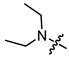	1	3.177	669.4 ± 56.4	355.3 ± 51.1	2
**4b**	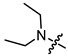	2	3.431	390.9 ± 23.1	99.5 ± 8.7	4
**4c**	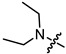	3	3.994	152.3 ± 12.1	30.5 ± 2.9	5
**5a**	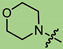	1	2.048	413.2 ± 35.1	5.0 ± 1	83
**5b**	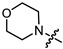	2	2.387	127.3 ± 15.2	8.5 ± 2.8	15
**5c**	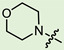	3	2.906	307.0 ± 24.1	5.8 ± 0.9	53
**6a**	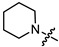	1	3.127	128.8 ± 9.8	41.9 ± 3.1	3
**6b**	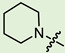	2	3.573	326.0 ± 45.6	14.6 ± 1.2	22
**6c**	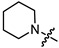	3	3.959	117.1 ± 11.1	39.0 ± 4.1	3
**7a**	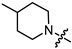	1	3.483	398.7 ± 4.8	109.1 ± 10.7	4
**7b**	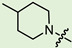	2	3.93	159.4 ± 17.1	6.2 ± 2.1	26
**7c**	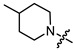	3	4.386	59.1 ± 4.8	19.9 ± 2.3	3
**8a**	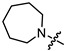	1	3.261	85.2 ± 5.6	61.3 ± 3.2	1
**8b**	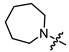	2	3.584	344.7 ± 24.6	97.6 ± 8.9	3
**8c**	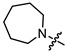	3	4.345	158.6 ± 12.4	34.2 ± 1.1	5
**9a**	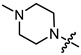	1	1.951	267.0 ± 16.5	13.9 ± 2.4	19
**9b**	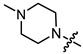	2	2.081	169.2 ± 12.1	17.2 ± 1.9	10
**9c**	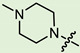	3	2.746	198.5 ± 13.6	5.1 ± 0.8	39
**10a**	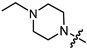	1	2.403	217.2 ± 18.9	57.7 ± 2.2	4
**10b**	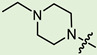	2	2.66	116.9 ± 11.7	5.9 ± 1.4	20
**10c**	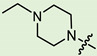	3	2.708	208.0 ± 21.4	9.8 ± 2.5	21
**11a**	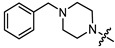	1	3.742	51.3 ± 4.9	21.6 ± 2.6	2
**11b**	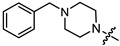	2	4.06	49.4 ± 5.6	46.8 ± 7.2	1
**11c**	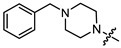	3	4.472	82.8 ± 7.9	25.1 ± 3.5	3
**12a**	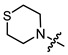	1	3.061	126.4 ± 10.5	69.6 ± 16.2	2
**12b**	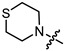	2	3.503	86.0 ± 7.4	96.3 ± 3.5	1
**12c**	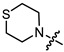	3	3.911	56.5 ± 3.8	75.3 ± 7.9	1
**13a**	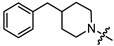	1	5.354	116.4 ± 13.4	21.6 ± 1.8	5
**13b**	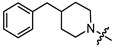	2	5.743	25.5 ± 11.4	14.9 ± 1.0	2
**13c**	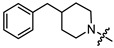	3	5.982	40.2 ± 5.1	7.3 ± 0.6	5
**14a**	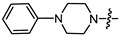	1	4.387	26.4 ± 3.8	4.6 ± 1.1	6
**14b**	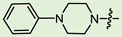	2	4.48	365.7 ± 25.8	8.1 ± 0.9	45
**14c**	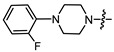	3	5.122	23.9 ± 8.7	12.0 ± 2.0	2
**15a**	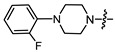	1	4.35	27.5 ± 3.8	2.7 ± 0.7	10
**15b**	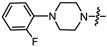	2	4.825	20.1 ± 12.1	8.2 ± 0.8	2
**15c**	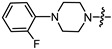	3	5.213	27.8 ± 7.4	14.7 ± 2.1	2
	Ribavirin			<4000	80.1 ± 13.5	50

^a^ CC_50_ is the median cytotoxic concentration, i.e., the concentration causing 50% cell death. ^b^ IC_50_ is the 50% inhibitory concentration, i.e., the concentration causing a 50% decrease in virus replication. ^c^ SI is the selectivity index, i.e., the CC_50_/IC_50_ ratio. CC_50_ and IC_50_ values are presented as the mean value ± standard deviation. All the values were calculated based on three independent experiments. logP, the octanol–water partition coefficient, was calculated based on the molecular descriptors of compounds using the plugin QikProp. Green means the activity of the compound is more high.

## Data Availability

Data is contained within the article and supplementary material.

## Supplementary Materials

The following supporting information can be downloaded at: https://www.mdpi.com/article/10.3390/ph15111390/s1, NMR 1H and 13C spectra of compounds **12**–**15a**–**c**; Molecular docking results; Molecular dynamics results.
